# Supplementary material for: Enhancing and shaping the immunogenicity of native-like HIV-1 envelope trimers with a two-component protein nanoparticle
Source: Nat Commun. 2019 Sep 19;10:4272. doi: 10.1038/s41467-019-12080-1 (PMC6753213; doi:10.1038/s41467-019-12080-1)
Supplement: Supplementary file 1 — Supplementary Information [file 41467_2019_12080_MOESM1_ESM.pdf]

Enhancing and shaping the immunogenicity of native-like HIV-1 envelope trimers with a two-component protein nanoparticle

Brouwer et al.

Supplementary Methods

Expression and Purification of Fabs for SPR

Fab fragments of selected antibodies (3BNC117, PGT122, PGT145, PGT151, 35O22, and 3BC315) were expressed and purified as follows. Plasmids encoding heavy chain and light chain of Fabs were transiently co-transfected (2:1) in HEK 293F suspension cells (Invitrogen, cat no. R79009) by the use of 293fectin transfection reagent (ThermoFisher Scientific). Supernatants were harvested 5 days post-transfection, centrifuged, filtered through 0.2 μm filters, and passed through an anti-human lambda/ kappa light chain affinity column (CaptureSelect Lambda LC / Kappa XL matrix, ThermoFisher Scientific) equilibrated with PBS at 4°C. Fabs were recovered from the affinity column by 0.1M Glycine (pH 3.0) and neutralized by 1M Tris-HCl (pH 8.0). Affinity purified Fabs were dialyzed in 1X PBS overnight at 4°C. Dialyzed Fabs were concentrated using Vivaspin filters with a 10 kDa molecular weight cutoff (GE Healthcare). The buffer was exchanged to 20mM sodium acetate (pH 5.6) for further purification by ion-exchange (IEX) followed by size exclusion chromatography (SEC) on AKTA FPLC instruments. For IEX, Fabs were loaded onto Mono S 5/50 GL (GE Healthcare) column and cation-exchanged with a gradient of Buffer A (sodium acetate; 20mM, pH 5.6) and Buffer B (sodium acetate; 20mM, KCl; 1.0M pH 5.6) from 0-50% at a flow rate of 1.0 mL min^{-1} over 60 min. Each Fab was eluted in accordance with its conductivity (3BNC117: 9.0 mS cm^{-1}; PGT122: 12 mS cm^{-1}; PGT145: 7.5 mS cm^{-1}; PGT151: 12 mS cm^{-1}; 35O22: 10 mS cm^{-1}; and 3BC315: 10 mS cm^{-1}); light chain monomers and dimers were eluted in later fractions. IEX fractions were analyzed on non-reducing SDS-PAGE gels (NuPAGE 4-12% Bis-Tris, Invitrogen), and fractions showing single band were selected and pooled. Any remnants of light chain were cleared by SEC. Fabs were then loaded on a SEC column (HiLoad 16/600 Superdex 200 pg, GE Healthcare) equilibrated in PBS. PBS was used as running buffer at a flow rate of 1.0 mL min^{-1}, fractions (0.5 ml) were collected, and Fab purity was assessed on non-reducing SDS-PAGE gels. SEC purified Fab fractions were pooled and protein concentration determined by the bicinchoninic (BCA) method. Purified Fab fragments were titrated in SPR experiments.

SOSIP mass determination by Field flow fractionation (FFF) and Multi-Angle Light Scattering (MALS)

As correct immobilization levels and stoichiometric analyses in SPR require the determination of the total mass, i.e., the peptidic mass plus that of the glycans, FFF-MALS was performed with BG505 SOSIP and ConM SOSIP to determine the molecular mass of the glycosylated trimers. Experiments were performed using an Eclipse DualTec module (Wyatt Technology Corporation) running on an Agilent 1260 Infinity II platform (Agilent Technologies). 50 μg of BG505 and ConM SOSIP were loaded onto a short channel unit assembled with a 350 μm wide spacer and regenerated cellulose

membrane (10 kDa molecular weight cutoff). Detector flow was set to 0.4 mL min^{-1} in all measurements, while cross-flow was set to 0.8 mL min^{-1}. The total run length was 100 min. The FFF system is coupled with an in-line MiniDawn Treos multiangle light scattering (MALS) detector, a quasi-elastic light scattering (QELS) detector and an Optilab T-reX refractive index (RI) detector (Wyatt Technology Corporation). Molecular mass values for SOSIP trimer samples were determined based on the analysis of the light scattering and UV280 absorbance data in Astra V software. Appropriate UV280 extinction coefficients were applied for each sample. The refractive index increment (dn dc^{-1}) was set to 0.168, a value experimentally determined for fully glycosylated Env[1]. The I53-50A extension with linker lacks glycans, and its total mass, $M_{\text{I53-50A}}$ = 24.0 kDa per protomer. The total mass of the likewise non-glycosylated B component monomer, $M_{\text{I53-50B}}$, = 18.2 kDa. Twelve pentamers of the monomer assemble into the NP.

The mass of SOSIP-I53-50NP is thus:

$$(1)\quad M_{\text{SOSIP-I53-50NP}} = 20 \cdot (M_{\text{SOSIP}} + 3 \cdot M_{\text{I53-50A}}) + 60 \cdot M_{\text{I53-50B}}$$

where M_{SOSIP} is the total mass of the trimer without the His tag as determined by FFF-MALS.

The determined molecular masses of BG505 SOSIP and ConM SOSIP are 350 and 320 kDa respectively; the calculated molecular mass of BG505 SOSIP-His and ConM-his are 355 and 325 kDa respectively and the calculated molecular mass of BG505 SOSIP-I53-50NP and ConM SOSIP-I53-50NP are 9500 and 8900 kDa respectively.

Fab and IgG binding to immobilized SOSIP trimers and SOSIP-I53-50NPs by SPR

Antibody binding to SOSIP trimers or SOSIP-I53-50NPs was analyzed by SPR. All SPR analyses were conducted on a BIAcore 3000 instrument at 25°C, with HBS-EP (10 mM HEPES, 150 mM NaCl, 3 mM EDTA, 0.005% surfactant P20) as running buffer. For immobilization of His-tagged SOSIP trimers and SOSIP-I53-50NPs, we chose the C1 sensor chip (GE Healthcare), which has a flat carboxy-methylated surface, instead of a regular dextran-coated chip. Thereby, we aimed to present both trimers and NPs without enmeshing them in dextran and in orientations such that they maximally expose all the native-trimer surfaces. The capture would occlude the base and C-terminus, in the case of the trimer, and a few His-tag-anchorage points on B components, with minimal distortion of surrounding trimers, in the case of the NPs. Anti-His antibody (GE Healthcare) was immobilized according to the manufacturer's instructions. Briefly, the C1 chip surface was pre-washed by injecting 100 mM glycine-NaOH (pH 12; with 0.3% triton X-100) twice for 1-2 min at a flow rate of 10 µL min^{-1} followed by 5-6 similar injections with HBS-EP running buffer. The chip surface was then activated by injecting a 1:1 mixture of NHS (N-Hydroxysuccinamide) and EDC (1-Ethyl-3-(3-dimethylaminopropyl) carbodiimide hydrochloride) for 15 min. Anti-His antibody (at 0.5 mg mL^{-1} in 10 mM sodium acetate, pH 4.5) was

injected for 20 min, and then ethanolamine for 5 min, yielding an immobilization of the capture antibody of 1500 RU. The flow rate was 10 µL min^{-1} throughout the amine coupling procedure.

SOSIP trimers were immobilized by His-tag capture at different levels as indicated by the R_L values given in the figure legends. SOSIP-I53-50NPs were immobilized to higher R_L values precisely in proportion to their non-Env content (36-39% higher for NPs than His-tagged trimers according to the variation in mass of the trimers of different genotypes) so as to get SOSIP amounts identical to those for the trimer alone. Since this equivalence is central to comparing the degrees of binding to trimers and NPs, we emphasize this point by giving normalized R_L values corresponding to the amount of SOSIP, either as trimers or presented on NPs.

Fabs or IgGs were injected at the concentrations indicated in the figures or figure legends. The flow rate was 30 µL min^{-1} for IgG. In order to minimize distortion of the kinetic modeling by transport limitation, Fabs were injected at the higher flow rate of 50 µL min^{-1}. In each cycle, the analyte (IgG or Fab) was allowed to associate for 300 s, and then dissociate for 600 s. At the end of each cycle, the chip surface was regenerated with single pulse of 10 mM Glycine (pH 2.0) at a flow rate of 75 µL min^{-1}. Each Fab was titrated from the highest concentration used (either 2 µM or 1 µM) in 2-fold dilutions until the signal was undetectable. To ascertain the specificity of binding, the signals of the control channel (which were <5% of the specific signals against SOSIP trimers and NPs) and of the zero-analyte injections were both subtracted. We also investigated whether naked I53-50NPs interacted non-specifically with the antibodies and demonstrated lack of Fab binding to immobilized naked I53-50NPs (Supplementary Fig. 5B).

The Fab titrations were performed in order to fit kinetic models to the binding data. In the ultimate modeling, the kinetic constants were obtained by global fits and the R_{max} values by local fits of the highest analyte concentrations. To investigate mass-transport limitation, however, global k_t ($RU \cdot M^{-1} \cdot s^{-1}$) fits, including global R_{max}, were also performed. In addition, the T values (= mean/s.e.m.) of the modeled k_t parameter were determined. If $k_t \geq 10^9$ ($RU \cdot M^{-1} \cdot s^{-1}$) and $T<10$ there is no significant mass-transport limitation. Indeed, the Fab binding (Supplementary Fig. 4C) in all cases easily passed the test for absence of mass-transport limitation. Therefore, the tabulated data in Table 3 are all kinetically limited and accordingly reliable. A simple Langmuir model fitted excellently to the binding data for all Fabs except for the binding of PGT151 Fab to the ConM SOSIP trimer. We therefore applied a conformational-change model, which fitted eminently to those binding data, showing significant parameter values for the conversion rate constants, k_f and k_b. In order to increase comparability, we applied the same model to the BG505 PGT151 Fab binding, although it improved the fit less and gave a lower forward-conversion-equilibrium constant (K_F) than for ConM (Table 3). We suggest that the known binding of PGT151 to only two of three potential epitopes on the trimer, an interaction that

distorts the third potential epitope, is compatible with both the kinetic and stoichiometric data in Table 3, which supports the model choice.

SOSIP trimer and SOSIP-I53-50NP binding to immobilized mAbs by SPR

In a converse approach, we studied the binding of SOSIP trimers and SOSIP-I53-50NPs to immobilized Abs. Affinity-purified goat anti-human IgG Fc (Bethyl Laboratories, Inc.) was amine-coupled to CM3 chips (GE Healthcare) by covalent amine coupling as described above. An immobilization level of 4000 RU of anti-Fc was achieved. This chip was chosen to allow sufficient anti-Fc immobilization and permit some flexibility through the low dextran matrix on the surface. Under such conditions some multipoint binding of SOSIP trimers and SOSIP-I53-50NPs may occur, as it would on a B-cell surface. Three flow cells were used for the capture of Env-specific Abs to R_L values of 320 ± 2.0 RU, whereas one flow cell with only anti-Fc Ab served as a control. To ascertain that SOSIP-I53-50NPs bind in an Env-specific manner, we demonstrated the lack of naked I53-50NP binding to immobilized IgG (Supplementary Fig. 5C).

Initially, binding to a panel of 13 NAbs and non-NAbs was analyzed for SOSIP trimers at 40 nM and SOSIP-I53-50NPs at 2 nM, i.e., with the same amount of Env per volume. The analytes were next titrated at the same ratio against 6 selected bNAbs. In contrast to the Fab binding, SOSIP trimer and SOSIP-I53-50NP binding to immobilized IgG fitted by simple Langmuir modeling (no model accounting for multi-point binding being feasible, see fitted curves in Fig. 4a) showed a severe mass-transport limitation ((MTL); $k_t < 10^8$ (RU · M^{-1} · s^{-1}) and T>10), particularly for the NP but in some cases also for the SOSIP trimers. These findings were expected since the mass of a trimer is 7-fold and the mass of the NP ~200-fold that of a Fab. The greater the molecular size, the slower the diffusion and the greater the tendency for mass-transport limitation. We therefore do not present kinetic modeling of the binding of SOSIP trimers and SOSIP-I53-50NPs to immobilized NAbs. To compare epitope accessibility, however, we calculated the number of molecules binding the immobilized IgG as follows:

$$(2) \quad n = \frac{R \cdot m \cdot A \cdot N_A}{M}$$

Where n is the number of macromolecules, R the response at 300 s (RU), m the mass bound per area and RU (g/(mm^2 · RU)), A the interactive area of the chip (mm^2), N_A Avogadro's constant (mol^{-1}) and M the molar mass of the macromolecule (either trimer or NP, (g mol^{-1})). This analysis brings the number of macromolecular ligation events for trimers and NPs onto a comparable basis by correcting for the greater mass and thereby greater signal for each bound NP.

Supplementary Note 1

Sequences

The leader sequence is shown in red, underlined amino acid sequences indicate the modified I53-50A scaffold fused to the SOSIP, the BamHI site is shown in blue. Mutations specific to the individual I53-50A variants are highlighted in green.

I53-50B.4PT1:

MNQHSHKDHETVRIAVVRARWHAEIVDACVSAFEAAMRDIGGDRFAVDVFDVPGAYEIPLH
ARTLAETGRYGAVLGTAFVVNGGIYRHEFVASAVINGMMNVQLNTGVPVLSAVLTPHNYD
KSKAHTLLFLALFAVKGMEAARACVEILAAREKIAAGSLEHHHHHH

I53-50A.1NT1:

MKMEELFKKHKIVAVLRANSVEEAIEKAVAVFAGGVHLIEITFTVPDADTVIKALSVLKEKG
AIIGAGTVTSVEQCRKAVESGAEFIVSPHLDEEISQFCKEKGVFYMPGVMTPTELVKAMKLG
HDILKLFPGEVVGPEFVKAMKGPFPNVKFVPTGGVDLDNVCEWFDAGVLAVGVGDALVEG
DPDEVREKAKEFVEKIRGCTEGSLEHHHHHH

BG505 SOSIP.v5.2-I53-50A:

MDAMKRGLCCVLLLCGAVFVSPSQEIHARFRRGARAENLWVTVYYGVPVWKDAETTLFCA
SDAKAYETKKHNVWATHCCVPTDPNPQEIHLENVTEEFNMWKNNMVEQMHTDIISLWDQS
LKPCVKLTPLCVTLQCTNVTNNITDDMRGELKNCSFNMTTELRDKKQKVYSLFYRLDVVQI
NENQGNRSNNSNKEYRLINCNTSAITQACPKVSFEPIPIHYCAPAGFAILKCKDKKFNGTGPCP
SVSTVQCTHGIKPVVSTQLLLNGSLAEEEVMIRSENITNNAKNILVQFNTPVQINCTRPNNNTR
KSIRIGPGQWFYATGDIIGDIRQAHCNVSKATWNETLGKVVKQLRKHFGNNTIIRFANSSGGD
LEVTTHSFNCGGEFFYCNTSGLFNSTWISNTSVQGSNSTGSNDSITLPCRIKQIINMWQRIGQA
MYAPPIQGVIRCVSNITGLILTRDGGSTNSTTETFRPGGGDMRDNWRSELYKYKVVKIEPLGV
APTRCKRRVVGRRRRRRAVGIGAVFLGFLGAAGSTMGAASMTLTVQARNLLSGIVQQQSNL
LRAPECQQHLLKLTVWGIKQLQARVLAVERYLRDQQLLGIWGCSGKLICCTNVPWNSSWSN
RNLSEIWDNMTWLQWDKEISNYTQIIYGLLEESQNQQEKNEQDLLALDGSGGSGGSGGSGGS
EKAAKAEEAARKMEELFKKHKIVAVLRANSVEEAIEKAVAVFAGGVHLIEITFTVPDADTVI
KALSVLKEKGAIIGAGTVTSVEQCRKAVESGAEFIVSPHLDEEISQFCKEKGVFYMPGVMTPT
ELVKAMKLGHTILKLFPGEVVGPQFVKAMKGPFPNVKFVPTGGVNLDNVCEWFKAGVLAV
GVGSALVKGTPDEVREKAKAFVEKIRGCTE

BG505 SOSIP.v5.2-I53-50A.1NT1:

MDAMKRGLCCVLLLCGAVFVSPSQEIHARFRRGARAENLWVTVYYGVPVWKDAETTLFCA
SDAKAYETKKHNVWATHCCVPTDPNPQEIHLENVTEEFNMWKNNMVEQMHTDIISLWDQS
LKPCVKLTPLCVTLQCTNVTNNITDDMRGELKNCSFNMTTELRDKKQKVYSLFYRLDVVQI
NENQGNRSNNSNKEYRLINCNTSAITQACPKVSFEPIPIHYCAPAGFAILKCKDKKFNGTGPCP
SVSTVQCTHGIKPVVSTQLLLNGSLAEEEVMIRSENITNNAKNILVQFNTPVQINCTRPNNNTR
KSIRIGPGQWFYATGDIIGDIRQAHCNVSKATWNETLGKVVKQLRKHFGNNTIIRFANSSGGD
LEVTTHSFNCGGEFFYCNTSGLFNSTWISNTSVQGSNSTGSNDSITLPCRIKQIINMWQRIGQA
MYAPPIQGVIRCVSNITGLILTRDGGSTNSTTETFRPGGGDMRDNWRSELYKYKVVKIEPLGV
APTRCKRRVVGRRRRRRAVGIGAVFLGFLGAAGSTMGAASMTLTVQARNLLSGIVQQQSNL

LRAPECQQHLLKLTVWGIKQLQARVLAVERYLRDQQLLGIWGCSGKLICCTNVPWNSSWSN
RNLSEIWDNMTWLQWDKEISNYTQIIYGLLEESQNQQEKNEQDLLALDGSGGSGGSGGSGGS
EKAAKAEEAARKMEELFKKHKIVAVLRANSVEEAIEKAVAVFAGGVHLIEITFTVPDADTVI
KALSVLKEKGAIIGAGTVTSVEQCRKAVESGAEFIVSPHLDEEISQFCKEKGVFYMPGVMTPT
ELVKAMKLGHDILKLFPGEVVGPEFVKAMKGPFPNVKFVPTGGVDLDNVCEWFDAGVLAV
GVGDALVEGDPDEVREKAKEFVEKIRGCTE

BG505 SOSIP.v5.2-I53-50A.1NT2:

MDAMKRGLCCVLLLCGAVFVSPSQEIHARFRRGARAENLWVTVYYGVPVWKDAETTLFCA
SDAKAYETKKHNVWATHCCVPTDPNPQEIHLENVTEEFNMWKNNMVEQMHTDIISLWDQS
LKPCVKLTPLCVTLQCTNVTNNITDDMRGELKNCSFNMTTELRDKKQKVYSLFYRLDVVQI
NENQGNRSNNSNKEYRLINCNTSAITQACPKVSFEPIPIHYCAPAGFAILKCKDKKFNGTGPCP
SVSTVQCTHGIKPVVSTQLLLNGSLAEEEVMIRSENITNNAKNILVQFNTPVQINCTRPNNNTR
KSIRIGPGQWFYATGDIIGDIRQAHCNVSKATWNETLGKVVKQLRKHFGNNTIIRFANSSGGD
LEVTTHSFNCGGEFFYCNTSGLFNSTWISNTSVQGSNSTGSNDSITLPCRIKQIINMWQRIGQA
MYAPPIQGVIRCVSNITGLILTRDGGSTNSTTETFRPGGGDMRDNWRSELYKYKVVKIEPLGV
APTRCKRRVVGRRRRRRAVGIGAVFLGFLGAAGSTMGAASMTLTVQARNLLSGIVQQQSNL
LRAPECQQHLLKLTVWGIKQLQARVLAVERYLRDQQLLGIWGCSGKLICCTNVPWNSSWSN
RNLSEIWDNMTWLQWDKEISNYTQIIYGLLEESQNQQEKNEQDLLALDGSGGSGGSGGSGGS
EKAAKAEEAARKMEELFKKHKIVAVLRANSVEEAIEKAVAVFAGGVHLIEITFTVPDADTVI
KALSVLKEKGAIIGAGTVTSVEQCRKAVESGAEFIVSPHLDEEISQFCKEKGVFYMPGVMTPT
ELVKAMKLGHDILKLFPGEVVGPEFVEAMKGPFPNVKFVPTGGVDLDDVCEWFDAGVLAV
GVGDALVEGDPDEVREDAKEFVEEIRGCTE

BG505 SOSIP.v5.2-I53-50A.1PT1:

MDAMKRGLCCVLLLCGAVFVSPSQEIHARFRRGARAENLWVTVYYGVPVWKDAETTLFCA
SDAKAYETKKHNVWATHCCVPTDPNPQEIHLENVTEEFNMWKNNMVEQMHTDIISLWDQS
LKPCVKLTPLCVTLQCTNVTNNITDDMRGELKNCSFNMTTELRDKKQKVYSLFYRLDVVQI
NENQGNRSNNSNKEYRLINCNTSAITQACPKVSFEPIPIHYCAPAGFAILKCKDKKFNGTGPCP
SVSTVQCTHGIKPVVSTQLLLNGSLAEEEVMIRSENITNNAKNILVQFNTPVQINCTRPNNNTR
KSIRIGPGQWFYATGDIIGDIRQAHCNVSKATWNETLGKVVKQLRKHFGNNTIIRFANSSGGD
LEVTTHSFNCGGEFFYCNTSGLFNSTWISNTSVQGSNSTGSNDSITLPCRIKQIINMWQRIGQA
MYAPPIQGVIRCVSNITGLILTRDGGSTNSTTETFRPGGGDMRDNWRSELYKYKVVKIEPLGV
APTRCKRRVVGRRRRRRAVGIGAVFLGFLGAAGSTMGAASMTLTVQARNLLSGIVQQQSNL
LRAPECQQHLLKLTVWGIKQLQARVLAVERYLRDQQLLGIWGCSGKLICCTNVPWNSSWSN
RNLSEIWDNMTWLQWDKEISNYTQIIYGLLEESQNQQEKNEQDLLALDGSGGSGGSGGSGGS
EKAAKAEEAARKMEELFKKHKIVAVLRANSVEEAIEKAVAVFAGGVHLIEITFTVPDADTVI
KALSVLKEKGAIIGAGTVTSVEQCRKAVESGAEFIVSPHLDEEISQFCKEKGVFYMPGVMTPT
ELVKAMKLGHDILKLFPGEVVGPQFVKAMKGPFPNVKFVPTGGVNLDNVCKWFKAGVLAV
GVGKALVKGKPDEVREKAKKFVKKIRGCTE

ConM SOSIP.v7-I53-50A:

MDAMKRGLCCVLLLCGAVFVSPSQEIHARFRRGARAENLWVTVYYGVPVWKDAETTLFCA
SDAKAYDTEKRNVWATHCCVPTDPNPQEIVLENVTENFNMWKNNMVEQMHTDIISLWDQS
LKPCVKLTPLCVTLNCTDVNATNNTTNNEEIKNCSFNITTELRDKKKKVYALFYKLDVVPID
DNNSYRLINCNTSAITQACPKVSFEPIPIHYCAPAGFAILKCNDKKFNGTGPCKNVSTVQCTHG

IKPVVSTQLLLNGSLAEEEIIIRSENITNNAKTIIVQLNESVEINCTRPNNNTRKSIRIGPGQWFY
ATGDIIGDIRQAHCNISRTKWNKTLQQVAKKLREHFNKTIIFNPSSGGDLEITTHSFNCGGEFF
YCNTSELFNSTWNGTNNTITLPCRIKQIINMWQRVGQAMYAPPIEGKIRCTSNITGLLLTRDG
GNNNTETFRPGGGDMRDNWRSELYKYKVVKIEPLGVAPTRCKRRVVERRRRRRAVGIGAVF
LGFLGAAGSTMGAASMTLTVQARNLLSGIVQQQSNLLRAPECQQHLLQLTVWGIKQLQARV
LAVERYLKDQQLLGIWGCSGKLICCTNVPWNSSWSNKSQDEIWDNMTWMEWDKEINNYTD
IIYSLIEESQNQQEKNEQELLALDGSGGSGGSGGSGGSEKAAKAEEAARKMEELFKKHKIVA
VLRANSVEEAIEKAVAVFAGGVHLIEITFTVPDADTVIKALSVLKEKGAIIGAGTVTSVEQCR
KAVESGAEFIVSPHLDEEISQFCKEKGVFYMPGVMTPTELVKAMKLGHTILKLFPGEVVGPQF
VKAMKGPFPNVKFVPTGGVNLDNVCEWFKAGVLAVGVGSALVKGTPDEVREKAKAFVEKI
RGCTE

AMC011 SOSIP.v8.1-I53-50A:

MDAMKRGLCCVLLLCGAVFVSPSQEIHARFRRGARAEQLWVTVYYGVPVWKDAETTLFCA
SDARAYDTEKRNVWATHCCVPTDPNPQEVVLENVTENFNMWKNNMVEQMHTDIISLWDQS
LKPCVKLTPLCVTLNCTDLRNATNTNATNTTSSSRGTMEGGEIKNCSFNITTSLRDKVQKEYA
LFYKLDVVPIKNDNTSYRLISCNTSVITQACPKVSFEPIPIHYCAPAGFAILKCNDKKFNGTGPC
TNVSTVQCTHGIRPVVSTQLLLNGSLAEEEVVIRSANFTDNAKIIIVQLNKSVEINCTRPNNNT
RKSIHIGPGRWFYTTGEIIGDIRQAHCNISGTKWNDTLKQIVVKLKEQFGNKTIVFNHSSGGDP
EIVMHSFNCGGEFFYCNSTQLFNSTWNDTTGSNYTGTIVLPCRIKQIVNMWQRVGQAMYAPP
IKGQIRCSSNITGLILIRDGGKNRSENTEIFRPGGGDMRDNWRSELYKYKVVKIEPLGIAPTRC
KRRVVQRRRRRRAVGIGAVSLGFLGAAGSTMGAASMTLTVQARQLLSGIVQQQNNLLRAPE
CQQHLLKDTHWGIKQLQARVLAVEHYLKDQQLLGIWGCSGKLICCTAVPWNTSWSNKSYN
QIWNNMTWMEWEREIDNYTSLIYTLIEDSQNQQEKNEQELLELDGSGGSGGSGGSGGSEKA
AKAEEAARKMEELFKKHKIVAVLRANSVEEAIEKAVAVFAGGVHLIEITFTVPDADTVIKALS
VLKEKGAIIGAGTVTSVEQCRKAVESGAEFIVSPHLDEEISQFCKEKGVFYMPGVMTPTELVK
AMKLGHTILKLFPGEVVGPQFVKAMKGPFPNVKFVPTGGVNLDNVCEWFKAGVLAVGVGS
ALVKGTPDEVREKAKAFVEKIRGCTE

ZM197M SOSIP.v5.2(519A, 568D, 570H, 585H)-I53-50A:

MDAMKRGLCCVLLLCGAVFVSPSQEIHARFRRGARMEQLWVTVYYGVPVWKEAKATLFCA
SDAKAYEKEVRNVWATHCCVPTDPNPQEIPLGNVTENFNMWKNDMADQMHEDIISLWDQS
LKPCVKLTPLCVTLNCSDATSNTTKNATNTNTTSTDNRNATSNDTEMKGEIKNCTFNITTEVR
DRKTKQRALFYKLDVVPLEEEKNSSSKNSSYKEYRLISCNTSTITQACPKVSFDPIPIHYCAPA
GYAILKCNNKTFNGTGPCHNVSTVQCTHGIKPVVSTQLLLNGSLAEEEIIIRSENLTDNTKTIIV
HLNESVEINCTRPNNNTRKSVRIGPGQWFFATGEIIGDIRQAHCNLSKSNWTTTLKRIEKKLKE
HFNNATIKFESSAGGDLEITTHSFNCRGEFFYCNTSGLFNSSLLNDTDGTSNSTSNATITLPCRI
KQIINMWQEVGRAMYASPIAGIITCKSNITGLLLTRDGGNKSAGIETFRPGGGNMKDNWRSE
LYKYKVVEIKPLGIAPTSCKRRVVERRRRRRAAGIGAVSLGFLGAAGSTMGAASVMLTVQA
RQLLSGIVQQQSNLLRAPECQQHMLQDTHWGIKQLQTRVLAIEHYLKDQQLLGLWGCSGKL
ICCTAVPWNTSWSNKSKDEIWDNMTWMQWDREIDNYTQVIYQLLEVSQNQQEKNENDLLA
LDGSGGSGGSGGSGGSEKAAKAEEAARKMEELFKKHKIVAVLRANSVEEAIEKAVAVFAGG
VHLIEITFTVPDADTVIKALSVLKEKGAIIGAGTVTSVEQCRKAVESGAEFIVSPHLDEEISQFC
KEKGVFYMPGVMTPTELVKAMKLGHTILKLFPGEVVGPQFVKAMKGPFPNVKFVPTGGVNL
DNVCEWFKAGVLAVGVGSALVKGTPDEVREKAKAFVEKIRGCTE

ConM SOSIP.v7-ferritin:

MDAMKRGLCCVLLLCGAVFVSPSQEIHARFRRGARAENLWVTVYYGVPVWKDAETTLFCA
SDAKAYDTEKRNVWATHCCVPTDPNPQEIVLENVTENFNMWKNNMVEQMHTDIISLWDQS
LKPCVKLTPLCVTLNCTDVNATNNTTNNEEIKNCSFNITTELRDKKKKVYALFYKLDVVPID
DNNSYRLINCNTSAITQACPKVSFEPIPIHYCAPAGFAILKCNDKKFNGTGPCKNVSTVQCTHG
IKPVVSTQLLLNGSLAEEEIIIRSENITNNAKTIIVQLNESVEINCTRPNNNTRKSIRIGPGQWFY
ATGDIIGDIRQAHCNISRTKWNKTLQQVAKKLREHFNKTIIFNPSSGGDLEITTHSFNCGGEFF
YCNTSELFNSTWNGTNNTITLPCRIKQIINMWQRVGQAMYAPPIEGKIRCTSNITGLLLTRDG
GNNNTETFRPGGGDMRDNWRSELYKYKVVKIEPLGVAPTRCKRRVVERRRRRRAVGIGAVF
LGFLGAAGSTMGAASMTLTVQARNLLSGIVQQQSNLLRAPECQQHLLQLTVWGIKQLQARV
LAVERYLKDQQLLGIWGCSGKLICCTNVPWNSSWSNKSQDEIWDNMTWMEWDKEINNYTD
IIYSLIEESQNQQEKNEQELLALDGSGDIIKLLNEQVNKEMQSSNLYMSMSSWCYTHSLDGAG
LFLFDHAAEEYEHAKKLIIFLNENNVPVQLTSISAPEHKFEGLTQIFQKAYEHEQHISESINNIV
DHAIKSKDHATFNFLQWYVAEQHEEEVLFKDILDKIELIGNENHGLYLADQYVKGIAKSRKS

Primers

BG505 sequence primers:

BG505 (forward 2)	GATGCCAAGGCTTACGAAAC
BG505 (forward 3)	TCTGTTTTATCGCCTGGATG
BG505 (forward 4)	TCACTAACAACGCTAAGAACATCC
BG505 (forward 5)	GCAGATCATTAATATGTGGCAGAG
BG505 (forward 6)	AATCTGCTGTCAGGGATCGT
BG505 (reverse 2)	ATCAGCTTTCCGCTGCAC
BG505 (reverse 3)	GGCTCGATCTTGACCACTTT
BG505 (reverse 4)	CGCAGTTGAAGCTGTGAGTT
BG505 (reverse 5)	GGTGGAGACCACAGGCTTAAT
BG505 (reverse 6)	AGACTGATCCCACAGGGAAA

ConM sequence primers:

ConM (forward 1)	GACGCCAAGGCCTACGATAC
ConM (forward 2)	CTGTTTTATAAACTGG
ConM (forward 3)	CCAACAACGCTAAGACAATC
ConM (forward 4)	GCAGATTATCAACATGTGGCAGGG
ConM (forward 5)	CTGCTGTCAGGCATTGTGCAGC
ConM (reverse 1)	CAGAGACTGATCCCACAGGG
ConM (reverse 1)	GCTGTGTGGAGACCACGGG
ConM (reverse 1)	CGCAGTTGAAACTATGTGTG
ConM (reverse 1)	GGGGTTCAATCTTGACCAC

General pPPI4 forward ORF sequence primer:

A5884 GGTGGAGGGCAGTGTAGTCT

General pPPI4 reverse ORF sequence primer:

A5890 CACCTTCCAGGGTCAAGGA

I53-50A reverse primer:

I53rev CGCCTCCAGCGAAGACTGCC

Mutation	SOSIP nomenclature					
	v4	v5	v6	v7	v8.1	
501C-605C	green	green	green	green	green	Sanders et al. 2013
559P	green	green	green	green	green	
R6	green	green	green	green	green	
.664	green	green	green	green	green	
156N+160N	green	green	green	green	green	
295N+301N	green	green	green	green	green	
332N+339N	green	green	green	green	green	
386N+392N	green	green	green	green	green	
448N	green	green	green	green	green	
64K/66R*	green	green	green	green	green	de Taeye et al. 2015
315Q	green	green	green	green	green	
316W	green	green	green	green	green	
535M	green	green	green	green	green	
543N	green	green	green	green	green	
73C-561C	red	green	green	green	green	Torrents de la Peña et al. 2017
49C-555C	red	red	green	red	red	
47D	red	red	red	green	green	Guenaga et al. 2015
49E	red	red	red	green	green	
65K	red	red	red	green	green	
106T	red	red	red	green	green	
165L	red	red	red	green	green	
429R	red	red	red	green	green	
432Q	red	red	red	green	green	
500R	red	red	red	green	green	
519S	red	red	red	red	green	Steichen et al. 2017
568D	red	red	red	red	green	
570H	red	red	red	red	green	
585H	red	red	red	red	green	

Supplementary Table 1. Nomenclature for novel stabilized SOSIP trimers, related to Fig. 3
All constructs have at least a v4 backbone. Green, present; red, not present. R6 refers to a hexa-arginine furin cleavage site. .664 refers to the truncation at residue 664. All residue positions are according to HxB2 numbering.
* The variant 64K is used for BG505 while the variant 66R is used for the rest of strains described here.

		Week 4						Week 6							Week 8		Week 12		Week 20	
		Control		Autologous		Heterlogous Tier 1B		Control		Autologous		Heterologous Tier 1A	Heterlogous Tier 1B		Autologous		Autologous		Autologous	
	Virus	MLV		ConM		ConS		MLV		ConM		SF162	ConS		ConM		ConM		ConM	
	Tier	-		1A		1B		-		1A		1A	1B		1A		1A		1A	
	Clade	-		-		-		-		-		B	-		-		-		-	
	Lab	AMC	DUMC	AMC	DUMC	AMC	DUMC	AMC	DUMC	AMC	DUMC	AMC	AMC	DUMC	AMC	DUMC	AMC	DUMC	AMC	DUMC
Immunogen	Rabbit ID																			
ConM SOSIP	2378	59	n.d.	117	n.d.	63	n.d.	75	<20	2776	707	23	150	36	1874	n.d.	1566	n.d.	709	n.d.
	2379	22	n.d.	<20	n.d.	<20	n.d.	<20	<20	<20	<20	<20	<20	<20	41	n.d.	94	n.d.	80	n.d.
	2380	<20	n.d.	<20	n.d.	<20	n.d.	<20	<20	188	70	<20	<20	<20	71	n.d.	47	n.d.	55	n.d.
	2381	21	n.d.	20	n.d.	<20	n.d.	<20	<20	3015	636	<20	<20	<20	1249	n.d.	2228	n.d.	1481	n.d.
	2382	<20	n.d.	152	n.d.	<20	n.d.	<20	<20	11815	2383	<20	<20	<20	4502	n.d.	1113	n.d.	556	n.d.
ConM SOSIP-ferritin	2388	<20	n.d.	93	n.d.	<20	n.d.	<20	<20	610	210	<20	<20	<20	181	n.d.	159	n.d.	134	n.d.
	2389	<20	n.d.	129	n.d.	<20	n.d.	<20	<20	5775	1177	<20	42	<20	7598	n.d.	2707	n.d.	1897	n.d.
	2390	<20	n.d.	66	n.d.	<20	n.d.	22	<20	5343	525	<20	156	<20	2292	n.d.	799	n.d.	283	n.d.
	2391	<20	n.d.	20	n.d.	<20	n.d.	<20	<20	1695	892	<20	39	<20	1778	n.d.	664	n.d.	920	n.d.
	2392	21	n.d.	178	n.d.	<20	n.d.	<20	<20	5727	489	<20	38	<20	3476	n.d.	1715	n.d.	795	n.d.
ConM SOSIP-I53-50NP	2393	22	n.d.	1335	n.d.	<20	n.d.	<20	<20	12276	4615	<20	125	24	14565	n.d.	2071	n.d.	672	n.d.
	2394	<20	n.d.	907	n.d.	<20	n.d.	<20	<20	16223	3959	21	215	53	18318	n.d.	2775	n.d.	3278	n.d.
	2395	31	n.d.	367	n.d.	<20	n.d.	<20	<20	18943	3177	<20	37	25	35307	n.d.	4873	n.d.	2049	n.d.
	2396	<20	n.d.	47	n.d.	186	n.d.	<20	<20	41223	5120	<20	93	27	16084	n.d.	2086	n.d.	2055	n.d.
	2397	<20	n.d.	818	n.d.	<20	n.d.	<20	<20	44545	33470	<20	1532	573	32875	n.d.	7426	n.d.	8764	n.d.

		Week 22																		
		Control		Autologous		Heterologous Tier 1A			Heterologous Tier 1B		Heterologous Tier 2									
	Virus	MLV		ConM		SF162	MW965	MN.3	ConS		25710-2.43	TRO.11	BJOX00200 0.03.2	X1632-S2-B10	Ce1176_A3	246-F3_C10_2	CH119.10	Ce70301021 7_B6	CNE55
	Tier	-		1A		1A	1A	1A	1B		2	2	2	2	2	2	2	2	2
	Clade	-		-		B	B	B	-		-	-	-	-	-	-	-	-	-
	Lab	AMC	DUMC	AMC	DUMC	AMC	DUMC	DUMC	AMC	DUMC	DUMC	DUMC	DUMC	DUMC	DUMC	DUMC	DUMC	DUMC	DUMC
Immunogen	Rabbit ID																		
ConM SOSIP	2378	45	<20	8118	4043	113	30	<20	476	82	<20	<20	<20	<20	<20	<20	<20	<20	<20
	2379	<20	<20	1703	1788	<20	945	<20	174	72	<20	<20	<20	<20	<20	<20	<20	<20	<20
	2380	<20	<20	12901	9042	<20	43	<20	422	126	<20	<20	<20	<20	<20	<20	<20	<20	<20
	2381	28	<20	17335	15481	48	199	34	1422	312	39	41	41	39	40	43	36	34	33
	2382	<20	<20	53063	20304	<20	1062	22	1311	391	<20	<20	<20	<20	<20	<20	<20	<20	<20
ConM SOSIP-ferritin	2388	<20	<20	4868	1999	284	2051	191	117	70	<20	<20	<20	<20	21	<20	26	<20	<20
	2389	<20	<20	115635	81961	151	1393	<20	2528	126	<20	<20	<20	<20	<20	<20	<20	<20	<20
	2390	<20	<20	92258	31387	114	2110	133	556	214	<20	<20	<20	<20	<20	<20	<20	<20	<20
	2391	<20	<20	87267	43325	488	2965	482	1658	539	<20	<20	<20	<20	<20	<20	<20	<20	<20
	2392	<20	<20	20207	10638	43	2396	49	310	91	<20	<20	<20	<20	<20	<20	<20	<20	<20
ConM SOSIP-I53-50NP	2393	<20	<20	56103	27174	145	278	139	957	283	<20	<20	<20	<20	<20	<20	<20	<20	<20
	2394	<20	<20	53337	33884	<20	449	<20	901	480	<20	<20	<20	<20	<20	<20	<20	<20	<20
	2395	<20	<20	121266	64360	<20	100	<20	847	197	<20	<20	<20	<20	<20	<20	<20	<20	<20
	2396	<20	23	40010	67908	<20	158	<20	650	207	<20	<20	<20	<20	<20	22	29	31	22
	2397	<20	<20	91310	74100	<20	279	<20	1960	712	<20	<20	<20	<20	<20	<20	<20	<20	<20

Supplementary Table 2. Midpoint neutralization titers at week 4, 6, 8, 12, 20 and 22 from rabbits that received ConM SOSIP, ConM SOSIP-ferritin or ConM SOSIP-I53-50NP tested against a panel of Env-pseudotyped viruses, related to Fig. 5c-5e

TZM-bl neutralization assays were performed either at the AMC or DUMC as indicated above each column. ID_{50} values, i.e. the serum dilution at which infectivity was inhibited by 50%, are shown and color coded: white = no neutralization, $ID_{50} < 20$; grey = very weak neutralization, $20 \leq ID_{50} \leq 40$; yellow = weak neutralization, $40 < ID_{50} \leq 100$; orange = moderate neutralization, $100 < ID_{50} \leq 1000$; red = strong neutralization, $1000 < ID_{50} \leq 10000$; purple = very strong neutralization, $ID_{50} > 10000$. Pink = cell toxicity observed in lowest dilution. MLV = murine leukemia virus (negative control). n.d. = not determined.

Week 22

		Control		Autologous		Heterologous Tier 1		
	Virus	MLV		BG505		SF162	MW965	MN.3
	Tier	-		2		1A	1A	1A
	Clade	-		A		B	B	B
	Lab	AMC	DUMC	AMC	DUMC	AMC	DUMC	Lab
Immunogen	Rabbit ID							
BG505 SOSIP	2163	<20	<20	3817	1305	20	166	<20
	2164	<20	<20	417	188	<20	86	<20
	2165	46	<20	4881	3220	65	254	72
	2166	<20	<20	159	95	<20	92	<20
	2167	58	<20	587	493	33	129	<20
	2168	36	<20	3143	1991	<20	36	<20
	2169	<20	<20	<20	<20	<20	35	<20
	2170	20	<20	1478	853	20	120	<20
BG505 SOSIP-I53-50NP	2171	<20	<20	561	365	34	772	60
	2172	<20	<20	22	<20	103	1657	64
	2173	<20	<20	92	65	31	877	<20
	2174	<20	<20	3936	1668	39	216	<20
	2175	<20	<20	<20	<20	<20	101	<20
	2176	<20	<20	2899	993	388	882	871
	2177	<20	<20	<20	<20	<20	75	<20
	2178	<20	<20	245	146	57	793	135

	RID_{50}					
Virus	BG505					
Mutant	241N	291T	241N+291T	611A	133aN+136aA	465N
Clade	A					
Lab	AMC					
Rabbit ID						
2163	0.01	0.01	0.01	0.30	0.38	0.24
2164	0.12	0.21	0.17	0.43	0.40	0.22
2165	0.01	0.01	0.01	0.34	0.28	0.35
2166	0.50	0.40	0.27	5.84	0.53	0.32
2167	0.09	0.06	0.16	0.62	0.49	0.47
2168	0.03	0.02	0.01	0.35	0.44	0.26
2169						
2170	0.58	1.46	0.35	2.56	0.05	1.31
2171	0.41	0.44	0.57	1.82	0.90	0.17
2172						
2173						
2174	0.48	0.25	0.27	0.76	0.48	0.37
2175						
2176	0.79	0.40	0.54	0.86	0.82	0.01
2177						
2178	0.63	0.46	0.43	1.54	0.81	0.89

Mutant/Parental
$RID_{50} < 0.25$
$RID_{50} \geq 0.25, \leq 0.5$
$RID_{50} > 0.5, \leq 2$
$RID_{50} > 2$

Supplementary Table 3. Midpoint neutralization titers at week 22 from rabbits that received BG505 SOSIP or BG505 SOSIP-I53-50NP tested against a panel of Env-pseudotyped viruses (left) and epitope mapping of BG505 neutralization (right), related to Fig. 5h and 6b

(left) TZM-bl neutralization assays were performed either at the AMC or DUMC as indicated above each column. ID_{50} values, i.e. the serum dilution at which infectivity was inhibited by 50%, are shown and color coded: white = no neutralization, $ID_{50} < 20$; grey = very weak neutralization, $20 \leq ID_{50} \leq 40$; yellow = weak neutralization, $40 < ID_{50} \leq 100$; orange = moderate neutralization, $100 < ID_{50} \leq 1000$; red = strong neutralization, $1000 < ID_{50} \leq 10000$. MLV = murine leukemia virus (negative control). (right) Relative ID_{50} (RID_{50}; ID_{50} BG505 mutant / ID_{50} parental BG505) shown for BG505 mutant viruses with mutations in the 241/289 glyan hole (241N, 291T, 241N+291T), the partially occupied glycan at 611 (611A), the V1V2 epitope (133aN + 136aA) and the C3/465 residue (465N). Epitope mapping was only performed for sera from rabbits that had an $ID_{50} > 100$. Color-coding is depicted in the top-right key.

Week 22

		Parental	RID50								
	Virus	ConS									
	Mutant	-	Y173A	N332A	N301A	G324A	W680R K683Q	V518L	T607S	T607K	T263K
	Clade	-									
	Lab	DUMC									
Immunogen	Rabbit ID										
ConM SOSIP	2378	200	0.63	1.87	0.89	0.66	1.71	0.98	1.16	1.60	1.00
	2379	259	0.47	0.86	1.27	0.71	1.40	0.90	1.15	1.80	0.92
	2380	210	0.65	1.90	2.48	0.55	1.35	1.11	1.66	2.67	1.24
	2381	432	0.30	1.50	0.83	0.42	1.40	0.93	1.16	1.89	0.28
	2382	485	0.68	2.54	0.91	0.13	2.07	1.64	2.33	6.01	1.72
ConM SOSIP-ferritin	2388	151	0.76	1.46	1.38	0.87	1.69	1.22	1.29	1.26	1.15
	2389	857	0.31	1.10	0.25	<0.02	0.51	0.54	0.79	1.45	0.97
	2390	198	0.53	1.20	0.95	0.26	1.01	0.70	1.03	1.94	1.05
	2391	454	0.31	1.27	0.82	0.20	1.07	0.85	1.13	2.28	1.01
	2392	185	0.56	1.55	0.57	0.13	1.67	0.82	1.12	4.46	1.05
ConM SOSIP-I53-50NP	2393	382	0.40	1.12	0.42	0.12	0.63	0.92	1.07	1.22	0.87
	2394	346	0.21	1.14	0.28	0.07	0.56	0.92	1.00	1.20	0.79
	2395	457	0.46	1.22	0.76	0.39	0.98	1.24	1.27	1.46	1.33
	2396	424	0.56	2.00	0.58	0.41	1.20	1.34	1.92	1.66	1.59
	2397	1808	0.29	1.31	0.51	0.07	1.28	1.02	1.59	1.67	1.19

Mutant/Parental
$RID_{50} < 0.25$
$RID_{50} \geq 0.25, \leq 0.5$
$RID_{50} > 0.5, \leq 2$
$RID_{50} > 2$

Supplementary Table 4. Epitope mapping of ConS neutralization from rabbits that received ConM SOSIP, ConM SOSIP-ferritin or ConM SOSIP-I53-50NP, related to Fig. 5d and 6d-6f

ConS neutralization titers (shown in Parental column) were determined using the TZM-bl neutralization assay. ID_{50} values, i.e. the serum dilution at which infectivity was inhibited by 50%, are shown and color coded: orange = moderate neutralization, $100 < ID_{50} \leq 1000$; red = strong neutralization, $1000 < ID_{50} \leq 10000$. Relative ID_{50} (RID_{50}; ID_{50} ConS mutant / ID_{50} parental ConS) shown for ConS mutant viruses with mutations in the V1V2 glycan (Y173A), V3 glycan (N332A, N301A, G324A), MPER (W680R, K683Q), fusion peptide (V518L) and gp120-gp41 interface (T607S, T607K, T263K). Color-coding is depicted in the top-right key.

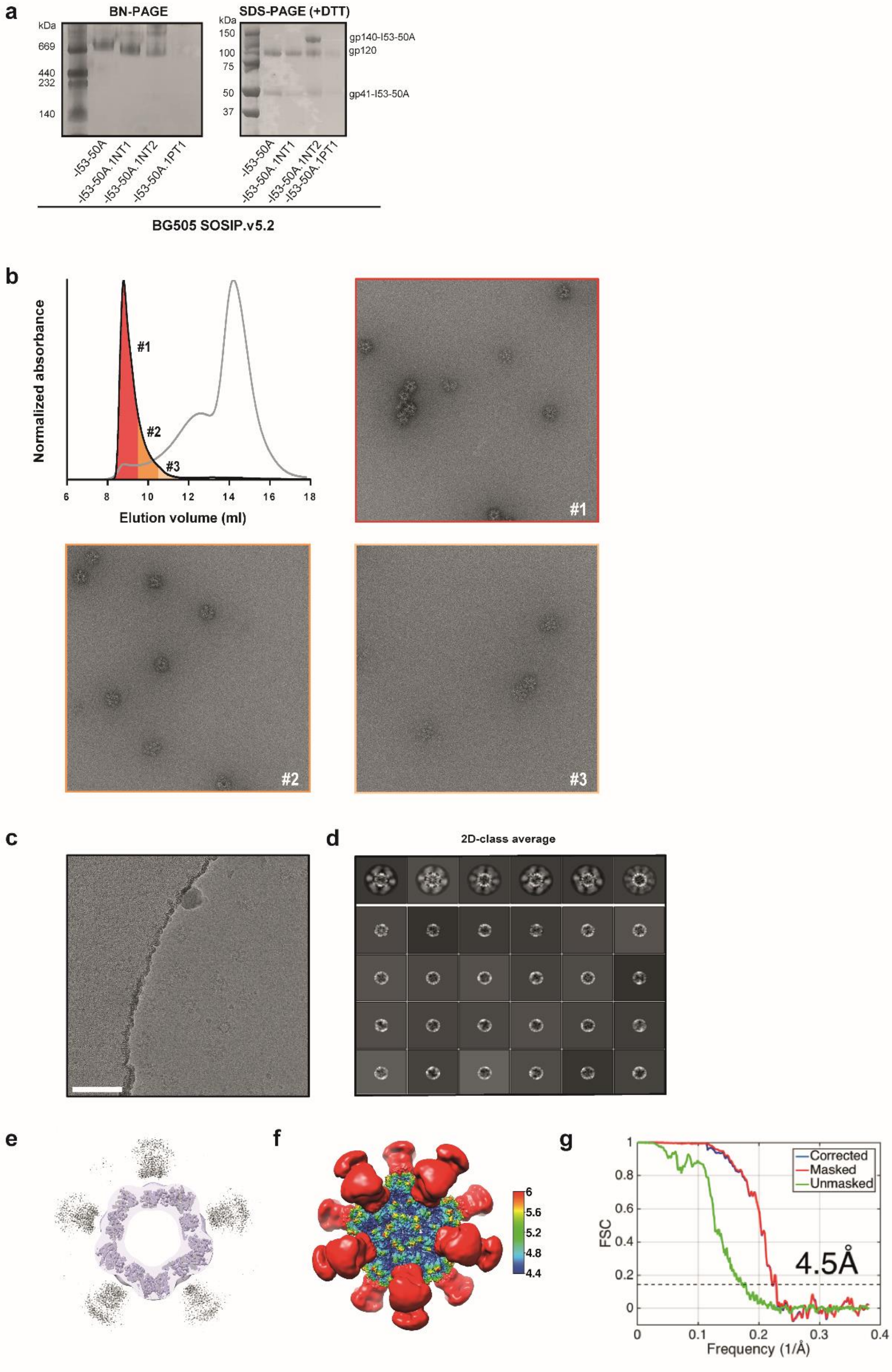

Supplementary Fig. 1. Screening of BG505 SOSIP.v5.2-I53-50A variants, characterization of BG505 SOSIP-I53-50NP assembly and additional cryo-EM data analysis, related to Fig. 1 and 2
a. BN-PAGE (left) and reducing SDS-PAGE (right) analysis of BG505 SOSIP.v5.2-I53-50A, -I53-50A.1NT1, -I53-50A.1NT2, and -I53-50A.1PT1. BG505 SOSIP.v5.2-I53-50A.1PT1 could not be purified to adequate yield

to allow clear bands on gel. Differences in band heights on BN-PAGE are likely caused by differences in the protein's isoelectric point. **b.** SEC profile of BG505 SOSIP-I53-50A.1NT1 (grey line) and the resulting BG505 SOSIP-I53-50NP (black line), after mixing with I53-50B.4PT1. Raw NS-EM micrographs are shown of the three pools annotated in NP SEC profile, with pool #1 (red: 8.5 ml-9.5 ml), pool#2 (orange: 9.5 ml-10.5 ml) and pool#3 (beige: 10.5 ml-11.5 ml). White bar corresponds to 200 nm. **c.** Representative contrast enhanced cryo-EM micrograph. White bar corresponds to 100 nm. **d.** Representative 2D class averages of the entire particle (top section) and I53-50NP core only (bottom section) showing the increased diversity of discovered classes achieved by excluding peripheral SOSIP density. **e.** 2D slice through the cryo-EM map (gray) within the tight mask (purple) that excludes peripheral BG505 SOSIP density used during 3D classification and refinement. **f.** Local resolution map of SOSIP-I53-50NP showing the color-coded drop differences in resolution (Å) between the NP core and the SOSIP trimers. **g.** Fourier shell correlation plot of independently refined half maps showing the resolution reported at FSC = 0.143 (dotted line). Source data are provided as a Source Data file.

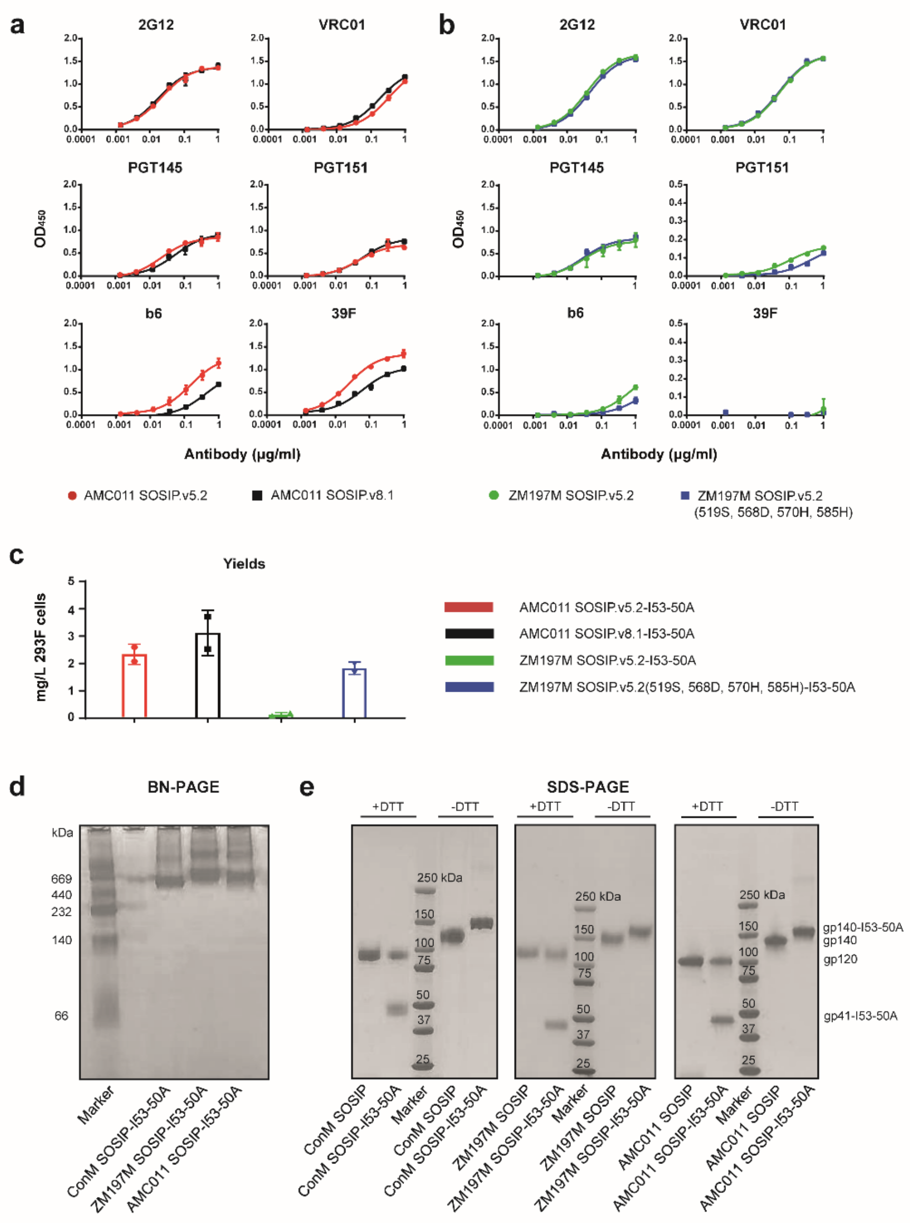

Supplementary Fig. 2. Antigenic characterization of an improved AMC011 and ZM197M SOSIP and biophysical characterization of ConM, AMC011, and ZM197M-SOSIP-I53-50A constructs, related to Fig. 3

a. Binding of bNAbs 2G12, VRC01, PGT145, PGT151 and non-NAbs b6 and 39F to immobilized AMC011 SOSIP.v5.2-His (red) and AMC011 SOSIP.v8.1-His (black) as analyzed by Ni-NTA ELISA. Shown are means

of two separate experiments with standard error of the mean. **b.** Binding of bNAbs 2G12, VRC01, PGT145, PGT151 and non-NAbs b6 and 39F to immobilized ZM197M SOSIP.v5.2-his (green) and ZM197M SOSIP.v5.2(519S, 568D, 570H, 585H)-his (blue) as analyzed by Ni-NTA ELISA. Shown are means of two separate experiments with standard error of the mean. **c.** Yields of AMC011 SOSIP.v5.2, AMC011 SOSIP.v8.1, ZM197M SOSIP.v5.2, and ZM197M SOSIP.v5.2(519S, 568D, 570H, 585H)-I53-50A. Shown are means of two individual transfections with standard deviations. **d.** BN-PAGE analysis of ConM, ZM197M, and AMC011 SOSIP-I53-50A. **e.** Reduced (+DTT) and non-reduced (-DTT) SDS-PAGE analysis of SOSIP-I53-50A versus the corresponding trimer with ConM SOSIP (left), ZM197M SOSIP (middle) and AMC011 SOSIP (right). Source data are provided as a Source Data file.

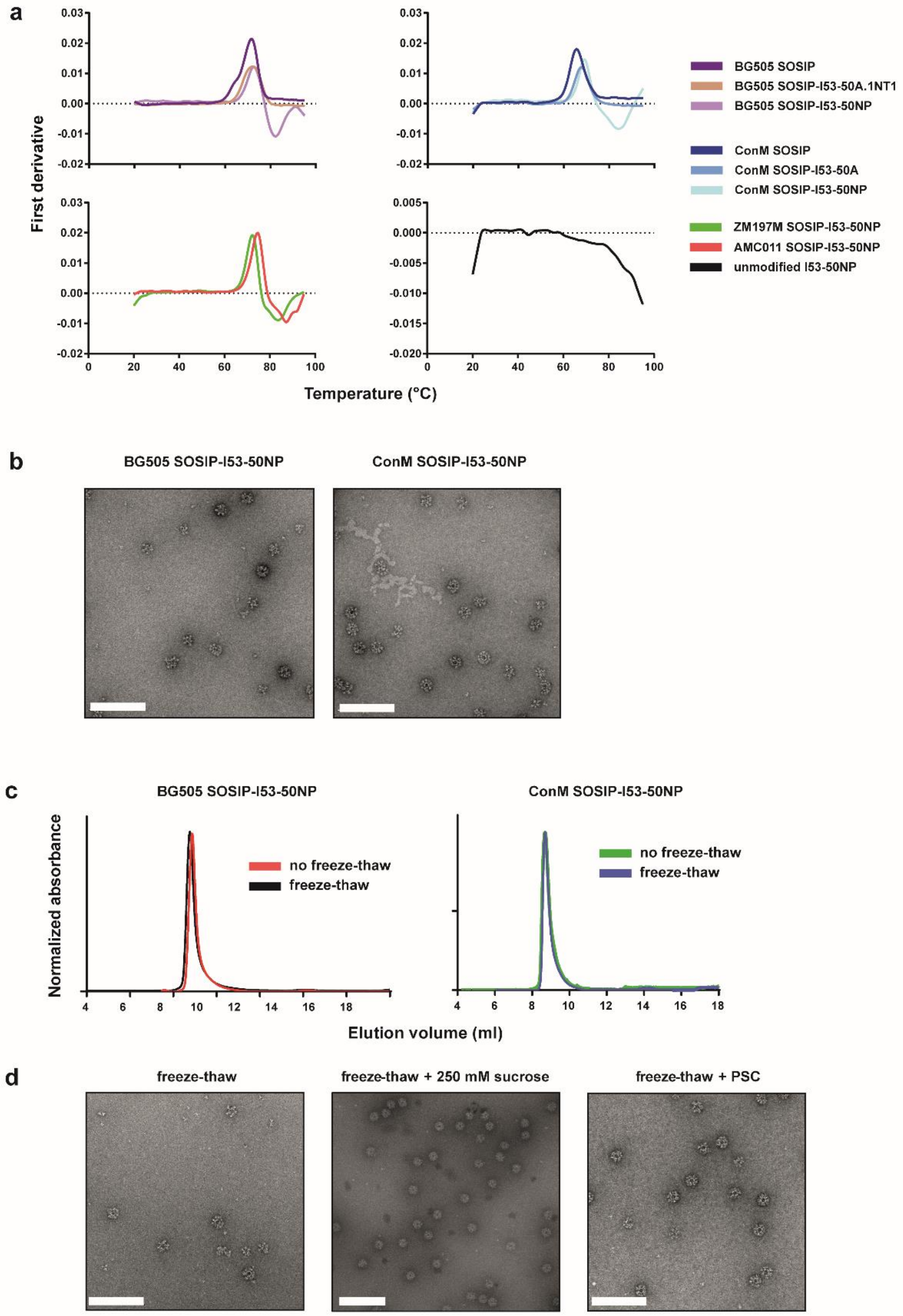

Supplementary Fig. 3. Thermostability of BG505, ConM, ZM197M, and AMC011 SOSIP-I53-50NPs and the effect of a single freeze-thaw cycle at -80°C on BG505, ConM, and AMC011 SOSIP-I53-50NPs, related to Fig. 1, 2, 3 and Table 1

a. NanoDSF curves of BG505, ConM, ZM197M and AMC011 SOSIP-I53-50NPs. Protein unfolding is measured as the ratio of tryptophan fluorescence 350 and 330 nm. Shown is the first derivative of this ratio with the T_m (temperature at the highest or lowest level of a peak or valley) determined as the ratio where 50% of the protein is unfolded. As the majority of tryptophans are surface exposed on the I53-50NP core, the ratio of 350/330 nm decreases upon denaturation leading to a negative first derivative as observed for the unmodified I53-50NP core. **b.** Raw NS-EM micrographs of BG505 and ConM SOSIP-I53-50NP after freeze-thawing at -80°C. White bar corresponds to 200 nm. **c.** Overlay of SEC profiles of BG505 and ConM SOSIP-I53-50NP before (red and green line) and after (black and blue line) freeze-thawing at -80°C. **d.** Raw NS-EM micrographs of AMC011 SOSIP-I53-50NP after freeze-thawing in the absence of a cryoprotectant (left) or in the presence of 250 mM sucrose (middle) or PSC (right). White bar corresponds to 200 nm. Source data are provided as a Source Data file.

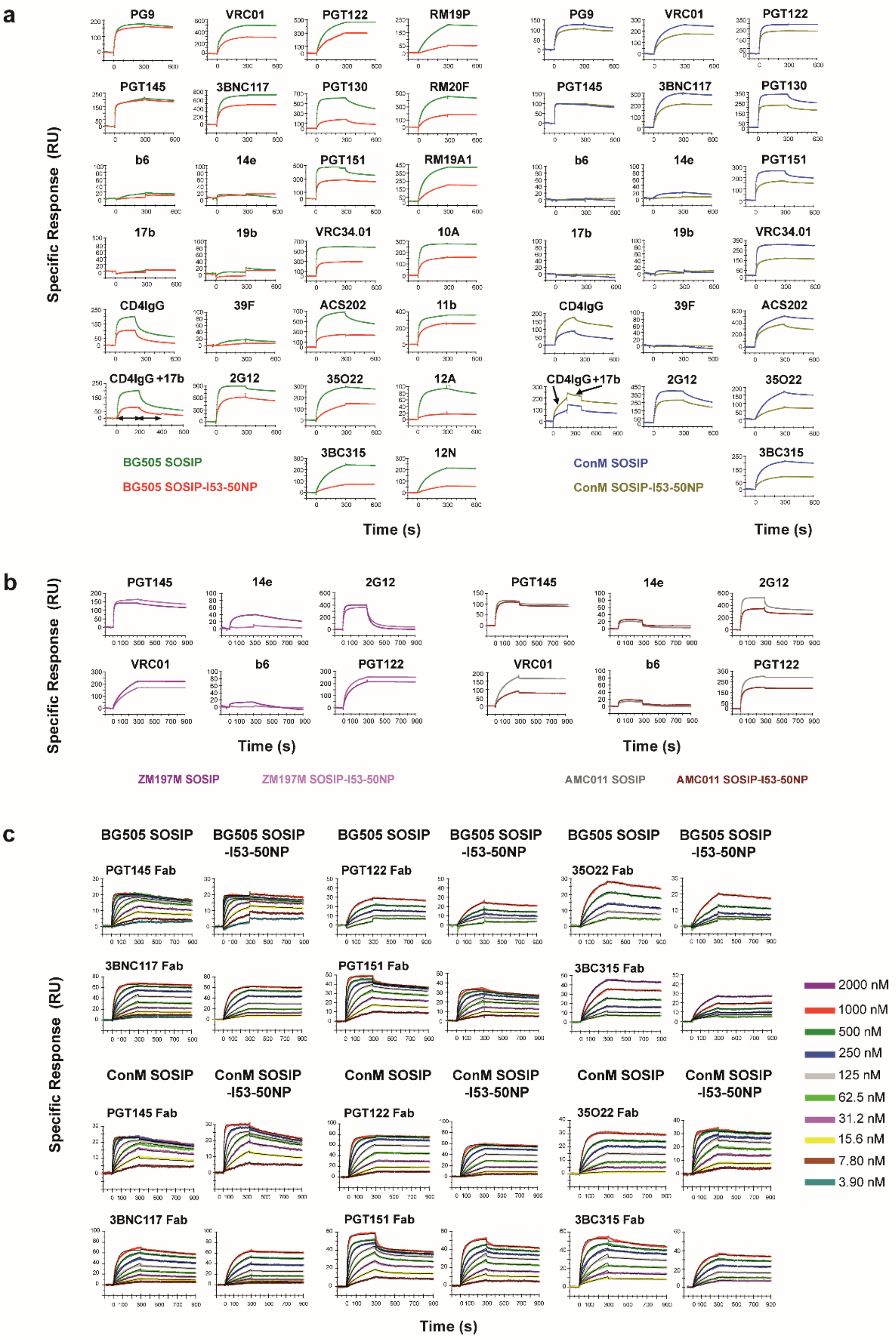

Supplementary Fig. 4. Binding of IgG and Fab to immobilized SOSIP-I53-50NPs and corresponding trimers, related to Fig. 4 and Table 3

a-c. The specific binding response in RU is plotted on the y -axis as a function of injection time on the x-axis, encompassing 300 s of association and 300 s (A) or 600 s (B, C) of dissociation. **a.** Binding of monoclonal Abs (Mabs), at 1 μM, indicated above each sensorgram to BG505 SOSIP-I53-50NP (left panel) and ConM SOSIP-I53-50NP (right panel) compared to trimers as indicated in the color code below. Apart from bNAbs, the non-

NAbs b6, 14e, 17b, 19b and 39F are tested, as well as CD4-IgG and lack of induction of the 17b CD4i epitope by CD4-IgG (bottom left sensorgram). Specifically, for BG505, macaque MAbs (RM19P, RM20F and RM19A1 (Cottrell et al. manuscript in preparation) and rabbit MAbs (10A, 11B, 12A and 12N)[2] isolated from BG505 SOSIP recipients were added to the analysis. The BG505 SOSIP trimer and NPs were immobilized to R_L values corresponding to constituent Env of 560 ± 12 RU. The ConM SOSIP trimer and NPs were immobilized to R_L values corresponding to constituent Env of 300 ± 0.23 RU. **b.** The sensorgrams show binding of bNAbs (PGT145, VRC01, 2G12 and PGT122) and non-NAbs (14e, b6) at 1 μM IgG to immobilized AMC011 and ZM197 SOSIP-I53-50NPs and trimers as indicated in the color code below. The trimers and NPs were immobilized to R_L values corresponding to 300 ± 0.23 RU of Env trimer. **c.** Each pair of sensorgrams shows the binding of Fabs to immobilized BG505 SOSIP-I53-50NP or ConM SOSIP-I53-50NP (right) compared to corresponding trimer (left). Fabs of the bNAbs indicated above the sensorgrams were titrated as in the color code to the right. Fitted curves in black are overlaid on the colored binding curves for each concentration. **a, c.** The lower degree of binding by IgG to NPs could be due to, first, a greater extent of bivalent binding to the epitopes when presented on the nanoparticles, thereby yielding lower signal per paratope bound; second, reduced accessibility of epitopes on the nanoparticle-bound trimer; or, third, reduced intrinsic affinity or number of antigenic epitopes on the nanoparticle-bound trimer. The first explanation, differential degrees of bivalency, implies a slower dissociation by IgG from the trimer on the nanoparticle than from the free trimer (if the latter dissociation is detectable, i.e., $k_{off} > 10^{-5}$ s^{-1}). This is indeed obvious for PGT130, PGT151, and in particular for ACS202. Although IgG clearly gives greater saturation on the epitopes on NP than free trimers in some cases, with different dissociation kinetics, we note that this explanation does not apply to the somewhat reduced binding by the functionally monovalent bNAb 2G12 to the trimers on NPs. Thus, the second explanation, steric clashes due to the bulk of the IgG molecule may also partly hinder access to epitopes in the NP context. Interestingly, neither the saturation through bivalency nor the steric clashes seem to apply to NAbs directed to the apex. The third explanation is largely refuted by a comparison of Fab binding to the trimer in the two contexts. Overall, the Fabs bind similarly to BG505 and ConM SOSIP in the two contexts with small variations in both directions (see also Table 3). Only the affinity of the PGT122 Fab for both the BG505 and ConM trimers on NPs is moderately reduced (~3-fold). Notably, the binding of Fabs differs considerably less than that of IgG between trimers on particles and trimers, again supporting bivalent binding or steric constraints for IgG on the particles.

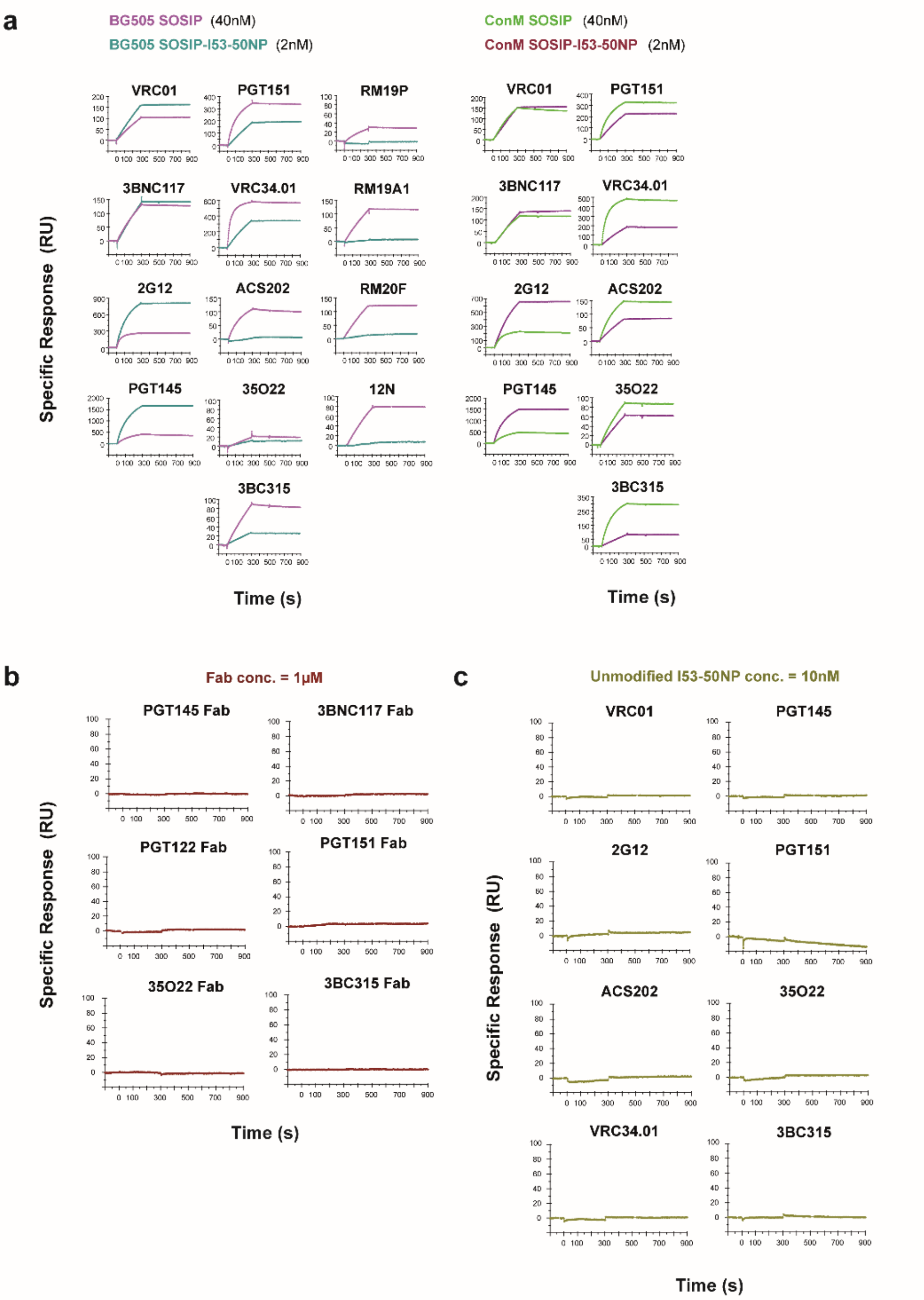

Supplementary Fig. 5. Binding of SOSIP-I53-50NPs and corresponding trimers to immobilized IgG and lack of binding by Fab and IgG to unmodified I53-50NPs, related to Fig. 4

a-c. The specific binding response in RU is plotted on the y-axis as a function of injection time on the x- axis, encompassing 300 s of association and 600 s of dissociation. **a.** The sensorgrams show the binding by BG505 SOSIP-I53-50NP (left panel) and ConM SOSIP-I53-50NP (right panel) compared to corresponding trimers as indicated in the color code above at the indicated concentrations. Concentrations correspond to equal amounts of Env per volume. bNAbs and MAbs were immobilized to R_L values of 320 ± 16 RU. **b.** The sensorgrams show the lack of binding of the Fabs (1 μM) to unmodified I53-50NPs as analyzed in Supplementary Fig. 4C and Table 3. The I53-50NPs were immobilized to R_L values of 210 ± 16 RU. **c.** The lack of binding of unmodified I53-50NPs to the immobilized bNAbs, as shown in Fig. 4a and (a), indicated above the sensorgrams is shown. Note the lack of binding although the I53-50NP concentration was 5 times higher than the highest concentration of SOSIP-I53-50NP shown in Fig. 4a. The bNAbs were immobilized to R_L values of 320 ± 2.0 RU.

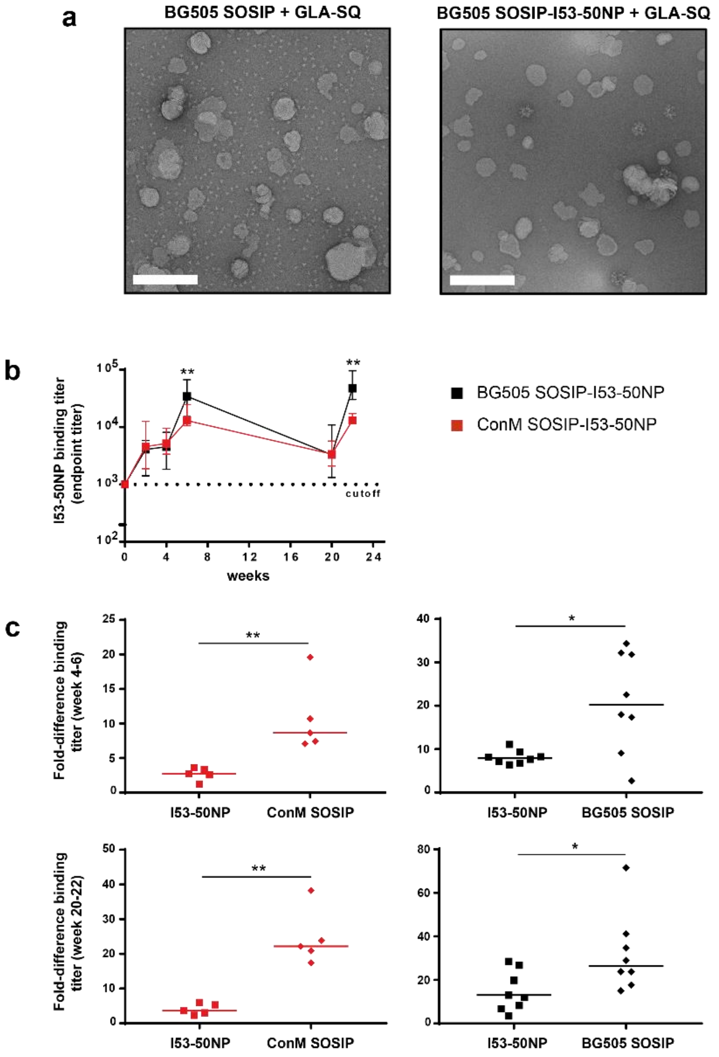

Supplementary Fig. 6. Influence of the adjuvant on BG505 SOSIP and BG505 SOSIP-I53-50NP integrity and the immunogenicity of I53-50NP, related to Fig. 5

a. Raw NS-EM micrographs showing that BG505 SOSIP (left) and BG505 SOSIP-I53-50NP (right) maintain their structural integrity in the presence of GLA-SQ. Trimers and NPs were mixed 1:1 with GLA-SQ and incubated for 30 min on ice prior to imaging. **b.** I53-50NP endpoint binding titers over time as measured by Ni-NTA ELISA with sera from rabbits that received BG505 SOSIP-I53-50NP (black squares, n=8 individual rabbits) or ConM SOSIP-I53-50NP (red squares, n=5 individual rabbits). Shown are medians with interquartile range. Statistical differences between two groups were determined using unpaired two-tailed Mann-Whitney U-tests (**: $p < 0.01$). Note that, in contrast to ConM-immunogen recipients, BG505-immunogen recipients received an extra boost at week 8, biasing the week 22 comparison. **c.** Plotted are the fold-differences of I53-50NP (squares) or SOSIP (rhombi) endpoint binding titers between week 4 and 6 (top panels) or week 20 and 22 (bottom panel). Titers were obtained by Ni-NTA ELISA with sera from rabbits that received ConM SOSIP-I53-50NP (red, n=5 individual rabbits) or BG505 SOSIP-I53-50NP (black, n=8 individual rabbits). Statistical differences between two groups were determined using unpaired two-tailed Mann-Whitney U-tests (*: $p < 0.05$; **: $p < 0.01$). Horizontal bars indicate the median. Source data are provided as a Source Data file.

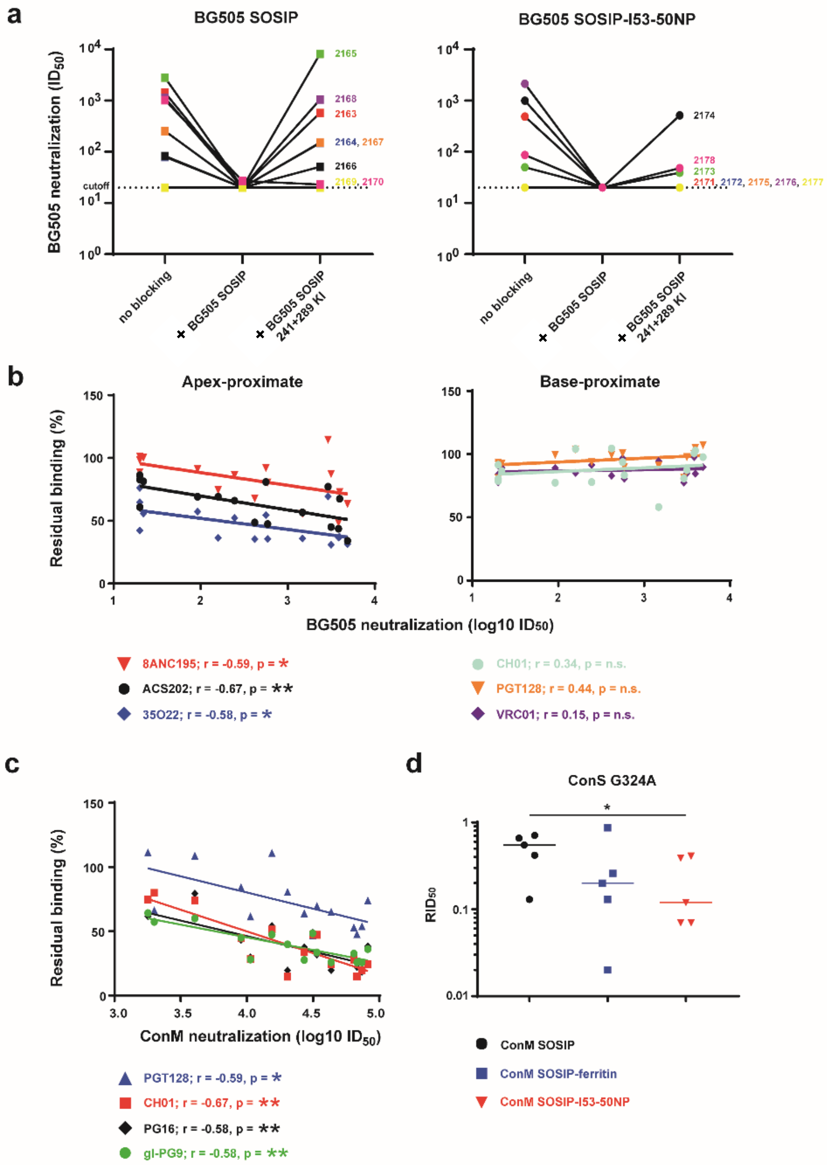

Supplementary Fig. 7. Epitope mapping of Ab-responses induced by rabbits immunized with BG505 and ConM immunogens, related to Fig. 6.

a. Midpoint autologous BG505 neutralization in the absence of a blocking agent (no blocking), with BG505 SOSIP added (+ BG505 SOSIP) or with BG505 SOSIP that has a glycan at position 241 and 289 (+ BG505 SOSIP 241+289 KI). The rabbit ID's of rabbits immunized with BG505 SOSIP (n=8 individual rabbits, left) or BG505 SOSIP-I53-50NPs (n=8 individual rabbits, right) are indicated. **b.** Correlation plot of BG505 neutralization *vs.* residual binding of 8ANC195, ACS202, 35O22, CH01, PGT128 and VRC01. The r and p values are shown for non-parametric Spearman correlations (n=16 individual rabbits; *: p<0.05; **: p< 0.01). **c.** Correlation plot of ConM neutralization versus residual binding of PGT128, CH01, PG16 or gl-PG9. The r and p values are shown for non-parametric Spearman correlations (n=15 individual rabbits; *: p<0.05; **: p< 0.01). **d.** The midpoint neutralization titers of ConS G324A mutant relative to the parental ConS virus is plotted (RID_{50} = ID_{50} against mutant virus/ ID50 against parental virus). Statistical differences between two groups (n=5 individual rabbits) were determined using unpaired two-tailed Mann-Whitney U-tests (*: p< 0.05). Horizontal bars indicate the median. See also Supplementary Table 4. Source data are provided as a Source Data file.

Supplementary References

1. Depetris, R. S. et al. Partial enzymatic deglycosylation preserves the structure of cleaved recombinant HIV-1 envelope glycoprotein trimers. J. Biol. Chem. 287, 24239–24254 (2012).

2. McCoy, L. E. et al. Holes in the Glycan Shield of the Native HIV Envelope Are a Target of Trimer-Elicited Neutralizing Antibodies. Cell Rep. 16, 2327–2338 (2016).
