# Supplementary material for: Enhancing and shaping the immunogenicity of native-like HIV-1 envelope trimers with a two-component protein nanoparticle
Source: Nat Commun. 2019 Sep 19;10:4272. doi: 10.1038/s41467-019-12080-1 (PMC6753213; doi:10.1038/s41467-019-12080-1)
Supplement: Supplementary file 3 — Reporting Summary [file 41467_2019_12080_MOESM3_ESM.pdf]

nature research

Corresponding author(s): Rogier W. Sanders
Neil P. King

Last updated by author(s): Aug 9, 2019

Reporting Summary

Nature Research wishes to improve the reproducibility of the work that we publish. This form provides structure for consistency and transparency in reporting. For further information on Nature Research policies, see Authors & Referees and the Editorial Policy Checklist .

Statistics

For all statistical analyses, confirm that the following items are present in the figure legend, table legend, main text, or Methods section.

n/a	Confirmed	
☐	☒	The exact sample size (*n*) for each experimental group/condition, given as a discrete number and unit of measurement
☐	☒	A statement on whether measurements were taken from distinct samples or whether the same sample was measured repeatedly
☐	☒	The statistical test(s) used AND whether they are one- or two-sided<br>*Only common tests should be described solely by name; describe more complex techniques in the Methods section.*
☒	☐	A description of all covariates tested
☐	☒	A description of any assumptions or corrections, such as tests of normality and adjustment for multiple comparisons
☐	☒	A full description of the statistical parameters including central tendency (e.g. means) or other basic estimates (e.g. regression coefficient) AND variation (e.g. standard deviation) or associated estimates of uncertainty (e.g. confidence intervals)
☐	☒	For null hypothesis testing, the test statistic (e.g. *F*, *t*, *r*) with confidence intervals, effect sizes, degrees of freedom and *P* value noted<br>*Give P values as exact values whenever suitable.*
☒	☐	For Bayesian analysis, information on the choice of priors and Markov chain Monte Carlo settings
☒	☐	For hierarchical and complex designs, identification of the appropriate level for tests and full reporting of outcomes
☒	☐	Estimates of effect sizes (e.g. Cohen's *d*, Pearson's *r*), indicating how they were calculated

Our web collection on statistics for biologists contains articles on many of the points above.

Software and code

Policy information about availability of computer code

Data collection	Leginon software was used for negative-stain and cryo-electron microscopy. 2D-class average data collection was performed using the Iterative MSA/MRA algorithm. Microsoft Excel was used to store raw data tables. The sic_axle code was used to dock SOSIP to I53-50A.
Data analysis	Generally Graphpad Prism 7.0 was used for data analysis. PyMOL 1.8.4.0. was used to analyze designs. Empower 3 software was used for glycan analysis. Appion software was used for NS-EM data processing. Relion/2.0, UCSF Chimera, RosettaRelax, EMRinger and MolProbity were used for cryo-EM data processing/model building.

For manuscripts utilizing custom algorithms or software that are central to the research but not yet described in published literature, software must be made available to editors/reviewers. We strongly encourage code deposition in a community repository (e.g. GitHub). See the Nature Research guidelines for submitting code & software for further information.

Data

Policy information about availability of data

All manuscripts must include a data availability statement. This statement should provide the following information, where applicable:

- Accession codes, unique identifiers, or web links for publicly available datasets
- A list of figures that have associated raw data
- A description of any restrictions on data availability

A reconstructed density map and refined coordinates of the I53-50NP core of BG505 SOSIP-I53-50NP have been deposited in the Electron Microscopy Database (EMD-20261) and the Protein Databank (6P6F). All other data supporting the findings in this manuscript are available from the corresponding authors (R.W.S. and N.P.K.) upon reasonable request. A static executable of the sic_axle program used to dock SOSIP to I53-50A is available upon request (neilking@uw.edu).

Field-specific reporting

Please select the one below that is the best fit for your research. If you are not sure, read the appropriate sections before making your selection.

- [x] Life sciences
- [ ] Behavioural & social sciences
- [ ] Ecological, evolutionary & environmental sciences

For a reference copy of the document with all sections, see nature.com/documents/nr-reporting-summary-flat.pdf

Life sciences study design

All studies must disclose on these points even when the disclosure is negative.

Sample size	No sample-size calculations were performed.
Data exclusions	No data was excluded from analyses.
Replication	All data points were repeated or performed in duplicate with the exception of several Abs in the competition ELISAs (although results are shown from 5/8 independent samples) due to the unavailability of the D7234 Ab.
Randomization	Allocation was random.
Blinding	Blinding was not relevant for our studies.

Reporting for specific materials, systems and methods

We require information from authors about some types of materials, experimental systems and methods used in many studies. Here, indicate whether each material, system or method listed is relevant to your study. If you are not sure if a list item applies to your research, read the appropriate section before selecting a response.

Materials & experimental systems

n/a	Involved in the study	
[ ]	[x]	Antibodies
[ ]	[x]	Eukaryotic cell lines
[x]	[ ]	Palaeontology
[ ]	[x]	Animals and other organisms
[x]	[ ]	Human research participants
[x]	[ ]	Clinical data

Methods

n/a	Involved in the study	
[x]	[ ]	ChIP-seq
[x]	[ ]	Flow cytometry
[x]	[ ]	MRI-based neuroimaging

Antibodies

Antibodies used	Env-specific antibodies were obtained from either Michel Nussenzweig, James Robinson, Dennis Burton, Peter Kwong, Mark Connors, John Mascola or William Olson through the NIH AIDS Research and Reference Reagent Program.
Validation	N.A.

Eukaryotic cell lines

Policy information about cell lines

Cell line source(s)	HEK293F cells were obtained from Invitrogen, cat no. R79007.<br>B cells were provided by Takayuki Ota and David Nemazee.
Authentication	Cell lines were validated by vendors or collaborators.
Mycoplasma contamination	All cell lines were tested negative for mycoplasma contamination
Commonly misidentified lines (See ICLAC register)	None.

Animals and other organisms

Policy information about studies involving animals; ARRIVE guidelines recommended for reporting animal research

Laboratory animals	New Zealand White Rabbits, 2.5-3kg, female.
Wild animals	No wild animals were used.
Field-collected samples	No field-collected samples were used.
Ethics oversight	All procedures performed in rabbits were done by Covance and approved by Denver PA IACUC Committee, #0135-016 and #0171-017.

Note that full information on the approval of the study protocol must also be provided in the manuscript.
